# Thermally Activated Fluxionality Accelerates Nonradiative Decay in Titania Nanoclusters

**DOI:** 10.1021/acs.jpclett.6c01707

**Published:** 2026-06-30

**Authors:** Miguel Recio-Poo, Stefan T. Bromley, Scott G. Sayres, Francesc Illas, Alexey V. Akimov, Ángel Morales-García

**Affiliations:** † Departament de Ciència de Materials i Química Física and Institut de Química Teòrica i Computacional (IQTCUB), 16724 Universitat de Barcelona, 08028 Barcelona, Spain; ‡ Institució Catalana de Recerca i Estudis Avançats (ICREA), 08010 Barcelona, Spain; § School of Molecular Sciences, 7864 Arizona State University, Tempe, Arizona 85287, United States; ∥ Biodesign Center for Applied Structural Discovery, Arizona State University, Tempe, Arizona 85287, United States; ⊥ Department of Chemistry, 12292 University at Buffalo, The State University of New York, Buffalo, New York 14260, United States

## Abstract

Photoactive nanoclusters are often assumed to exist as a single isomer corresponding to the lowest-energy local minimum structure. However, at finite temperatures, multiple configurations may be thermally accessible. Using ab initio molecular dynamics combined with time-dependent density functional theory, we identify a fluxional mode associated with low-energy structural rearrangement that can directly influence excited-state dynamics in a model titania nanocluster. This thermally driven mode reshapes the low-energy excited-state manifold, producing a broader, bimodal S_1_ excitation-energy distribution. Nonadiabatic dynamics reveals two distinct consequences. Relaxation toward S_1_ accelerates because fluxionality changes the energetic end point of the decay pathway, whereas ground-state recombination accelerates through enhanced sampling of strongly coupled S_0_–S_1_ configurations. These findings establish local coordination fluxionality as a structural mechanism for reshaping excited-state landscapes in oxide nanoclusters, with implications for tuning nonradiative decay and charge recombination pathways in photocatalytic metal oxides.

## Graphical abstract

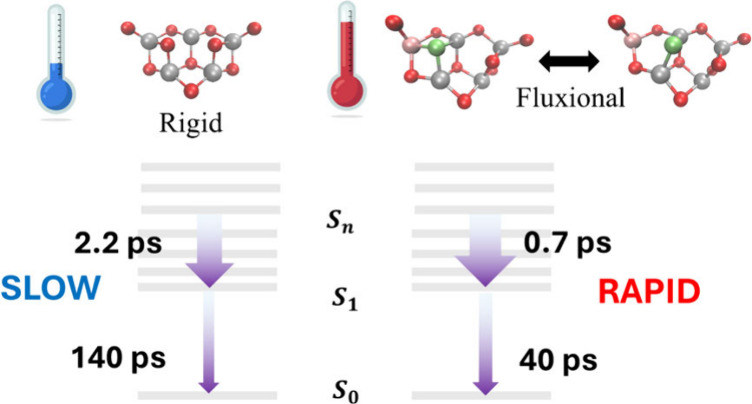

Understanding how atomic structure governs excited-state dynamics is central to the rational design of photoactive materials for photocatalysis, photovoltaics, light harvesting, and excitonic transport.^1–3^ This connection is usually discussed in terms of well-defined structural motifs, defects, surfaces, or coordination environments that control the localization, energy, and coupling of the electronic states.^4–7^ These structural descriptors, however, are not necessarily static. Systems in which structure, finite size, and thermal motion can be controlled or sampled explicitly are therefore particularly valuable for examining how nuclear dynamics affect excited states. Atomically precise nanoclusters (NCs) are especially useful in this context because they bridge molecular and extended material regimes, with electronic properties that depend sensitively on size, composition, and geometry.^8^ Rather than being reducible to a single optimized geometry, a NC may sample several competing configurations (e.g., metastable local minima) at a finite temperature, each contributing differently to its electronic properties.^9,10^

This ensemble perspective is especially important for fluxional clusters, which often possess shallow potential-energy landscapes with multiple low-lying minima connected by accessible pathways. Their finite-temperature behavior can involve thermally activated interconversion between competing structural motifs,^9,10^ ranging from nearly rigid, solid-like dynamics to nonrigid, liquid-like, or “slush-like” regimes as the temperature or internal energy increases. In some cases, distinct structural forms can coexist dynamically over a finite interval of internal energy or temperature, with the system fluctuating among regions of phase space associated with different structural character.^11,12^ Related effects are now well established in subnanometer metal NCs, particularly in catalysis, where the global minimum may describe only part of the relevant chemistry.^13,14^ Under realistic conditions, the relevant structure of a NC is often better viewed as a thermally weighted ensemble shaped by temperature, pressure, adsorbates, and interconversion among low-energy isomers.^14,15^ For instance, studies of H_2_ adsorption and dissociation on gold nanoclusters have shown that catalytic activity cannot always be inferred from the lowest-energy structure alone, because low-lying isomers and structural transformations can provide more favorable reaction pathways under finite-temperature conditions.^16^ Work on larger gold nanoparticles similarly showed that structural flexibility and adsorbate-induced distortions play a key role in H_2_ bonding and dissociation.^17^ Beyond catalysis, ultrafast experiments on small noble-metal clusters show that nuclear motion and the electronic response can be coupled on femtosecond to picosecond time scales, illustrating that structural dynamics in finite clusters may occur on time scales relevant to electronic relaxation.^18^ Neumark and co-authors also reported the isomer distribution of cluster oxides (e.g., Ti_2_O_4_ and Zr_2_O_4_) through slow photoelectron velocity-map imaging.^19^

The consequences of fluxionality for electronically excited states remain much less explored, particularly in oxide nanoclusters, where photoinduced charge separation, relaxation, and recombination pathways are highly sensitive to local metal–oxygen coordination and bond distortions.^20,21^ Atomically precise oxide nanoclusters are useful molecular-scale models of photoactive metal oxides because they allow finite stoichiometries, local coordination motifs, and thermally accessible nuclear configurations to be explicitly defined and sampled.^22,23^ Previous work has shown that oxide nanoclusters can exhibit premelting or conformationally fluxional dynamics, including interconversion between nearly isoenergetic motifs in MgO clusters and temperature-induced conformational switching in nanosilicate clusters.^24,25^ In the latter case, finite-temperature anharmonic motion and premelting dynamics were shown to alter calculated vibrational spectra relative to static harmonic descriptions, demonstrating that thermally induced structural fluxionality can lead to direct spectroscopic signatures. For excited states, this raises the related question of whether thermally activated structural motion can act as a control coordinate that reshapes excitation energies, state character, nonadiabatic couplings, and ultimately excited-state dynamics.

Temperature is central to this question because it determines which regions of the potential-energy landscape are dynamically accessible and therefore which structural motifs contribute to the excited-state ensemble. This is particularly relevant for titania systems, where temperature-dependent vibrational and configurational effects can alter thermodynamic stability, hydration, and excited-state relaxation pathways.^22,26^ Titanium dioxide (TiO_2_) is a prototypical photoactive oxide, widely studied for its stability, abundance, and relevance to photocatalysis.^27–29^ Small (TiO_2_)_*n*_ (*n* = 1–8) NCs provide molecular-scale models for probing TiO_2_ photophysics without the additional complexity of extended defects, surfaces, and long-range structural constraints. Their electronic excitations typically involve redistribution from O 2p-derived valence states to Ti 3d-derived acceptor states, making excitation energies, state character, and nonadiabatic couplings highly sensitive to local coordination and Ti–O bond distortions. In a recent combined experimental and nonadiabatic molecular dynamics (NA-MD) study of size-selected (TiO_2_)*_n_* NCs, we identified two dynamical regimes: (i) subpicosecond relaxation within the excited-state manifold and (ii) slower recombination to the ground state, whose size-dependent time scales reflect the combined influence of energy gaps, nonadiabatic couplings, densities of states, and finite-temperature nuclear motion.^22^ Similar sensitivity to structural and environmental effects has also been reported for hydrated titania clusters.^21^

For nearly all NCs examined in these studies, the observed relaxation and recombination trends could be rationalized using a dominant low-energy isomeric motif as the structural reference. The (TiO_2_)_5_ NC, however, stood out as an exception because it displayed structural fluxionality and broke the otherwise monotonic trends observed across the series.^22^ Consistent with this behavior, previous basin-hopping searches identified several low-lying (TiO_2_)_5_ isomers within a narrow energy window, suggesting a shallow potential-energy landscape with thermally accessible structural motifs.^30^ Here, we revisit this anomalous NC to determine how finite-temperature fluxionality reshapes its excited-state landscape and modifies relaxation and ground-state recombination dynamics. By combining finite-temperature *ab initio* molecular dynamics (AIMD), time-dependent density functional theory (TD-DFT) excitation analysis, and NA-MD, we identify a localized Ti–O coordination switch that becomes active at 300 K, modulates the low-energy excited-state manifold, and alters nonradiative decay through changes in excitation-energy distributions and nonadiabatic coupling statistics.

To investigate the relationship between the structural fluxionality of the (TiO_2_)_5_ NC and its excited-state properties, we conduct finite-temperature AIMD combined with TD-DFT calculations, starting from representative low-energy cluster geometries. Simulations are performed at 100 and 300 K in order to distinguish ordinary vibrational fluctuations within a local basin from thermally activated sampling of alternative structural motifs. Three low-lying structures are considered and hereafter termed isomers I–III (Figure 1a), with relative energies of 0.00, 0.02, and 0.08 eV, respectively. Isomer I corresponds to the fluxional motif, isomer II to the rigid structure previously studied elsewhere,^22^ whereas isomer III to an additional low-energy motif structurally related to configurations transiently accessed by isomer I. All three NCs^29^ consist of compact networks of edge- and corner-sharing TiO_*x*_ polyhedra but differ in symmetry and local connectivity. Isomer I contains a structurally soft Ti–O subunit that becomes activated at finite temperature. In contrast, isomer II adopts a more constrained framework-like structure that remains quasi-rigid, while isomer III provides a reference structure for the elongated local coordination motif sampled transiently by isomer I.

**Figure 1:**
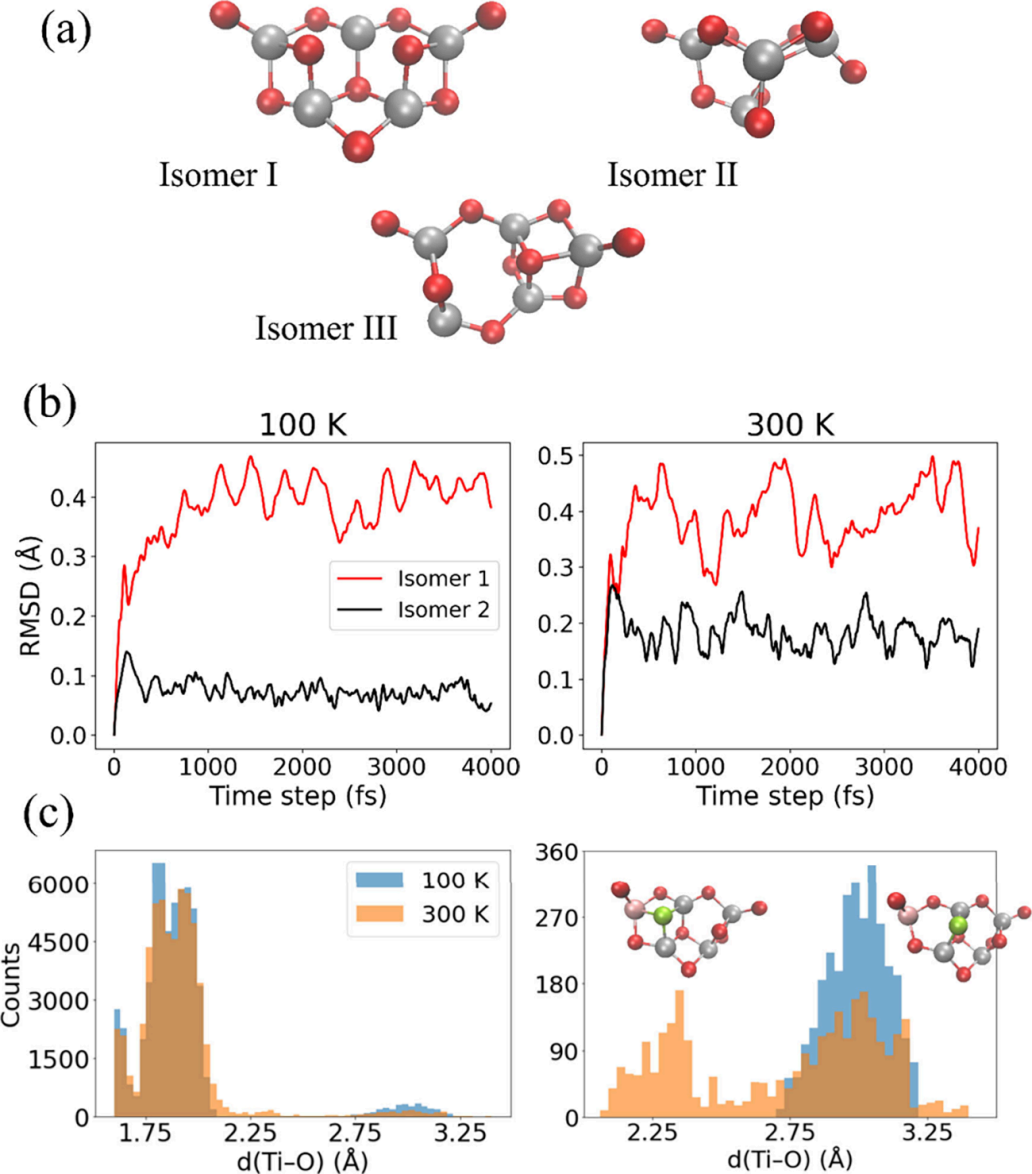
(a) Structures of the three low-lying (TiO_2_)_5_ isomers considered in this work, with calculated relative energies of 0.00, 0.02, and 0.08 eV for isomers I–III, respectively.^29^ Gray and red spheres denote Ti and O atoms, respectively. Isomer I corresponds to the fluxional motif, isomer II to the rigid reference structure, and isomer III to a structurally related low-energy motif. (b) Root-mean-square deviations (RMSD) of atomic positions along AIMD trajectories for isomers I and II at 100 K (left) and 300 K (right), after removal of overall translation and rotation. (c) Distribution of all Ti–O nearest-neighbor distances sampled along the AIMD trajectories at 100 and 300 K (left). Magnified histograms of the Ti_flux_–O_flux_ coordination distance for isomer I at 100 and 300 K (right). Insets show representative NC geometries sampled at short and long Ti_flux_–O_flux_ distances; the highlighted Ti_flux_ and O_flux_ atoms are colored pink and lime, respectively.

Ground-state electronic structure and AIMD simulations are conducted using the CP2K^31^ code. Exchange-correlation effects are described using the PBE functional^32^ and the MOLOPT Gaussian basis sets.^33^ The core electrons are described using GTH pseudopotentials. In addition, the D3 dispersion correction is used.^34^ AIMD trajectories are generated in the *NVT* ensemble at 100 and 300 K using a Nosé-Hoover thermostat,^35^ with a thermostat time constant of 100 fs. A time step of 1 fs is used in the MD runs for a total simulation time of 4 ps, with the first 1 ps discarded as equilibration. Configurations extracted from the production trajectories are used to represent the finite-temperature structural ensembles. Structural mobility is quantified using the root-mean-square deviation (RMSD) of configurations after optimal alignment with each of the three reference isomers, together with selected Ti–O distance distributions. Collective finite-temperature motion modes are further characterized using a velocity-covariance mode analysis following the Strachan approach.^36^

Vertical excitation energies are computed using linear-response TD-DFT as implemented in CP2K, with the range-separated CAM-B3LYP functional.^37^ For each sampled configuration, the lowest 20 singlet excited states are computed, and selected excited-state characters are analyzed using natural transition orbitals (NTOs). NA-MD calculations are conducted using the Libra package.^38,39^ The neglect-of-back-reaction approximation (NBRA)^28,40,41^ is used, according to which nuclei are propagated on the ground-state PES, and their motion is not affected by changes in the electronic state during photoexcitation or excited-state decay. Population dynamics are followed using the fewest switches surface-hopping (FSSH) algorithm^42^ and its combinations with the modified simplified decay of mixing (mSDM) approach,^43^ which introduces electronic decoherence corrections. This comparison allows us to assess the influence of nuclear motion-induced decoherence on relaxation and ground-state recombination. NA-MD trajectories are propagated for 3 ps using a time step of 1 fs. Further computational details are provided in section S1 of the Supporting Information.

We analyze both relaxation within the excited-state manifold and recombination to the ground state. Following the Kasha rule^44^ and consistent with our recent study of size-selected (TiO_2_)_*n*_ NCs, the dynamics is treated as a two-step process: relaxation of initially populated higher excited states toward the low-energy excited-state manifold, approaching S_1_, followed by slower nonradiative decay to S_0_. In the simulations, the relaxation time is extracted from the decay of the average excitation energy whereas the recombination time is obtained from the growth of the S_0_ population.

We begin by analyzing finite-temperature AIMD trajectories of the low-energy (TiO_2_)_5_ isomers shown in Figure 1a. Isomer II, previously identified as the rigid reference structure, remains close to its optimized geometry throughout thermal sampling. Geometry optimization of representative snapshots from this trajectory consistently relaxes back to the same minimum, indicating that the sampled distortions correspond to small-amplitude motion within a stiff local basin of the potential-energy surface (PES). In contrast, isomer I displays larger configurational fluctuations. Most snapshots extracted from this trajectory relax back to the optimized isomer I structure; however, selected highly distorted configurations instead relax to isomer III. This indicates that isomer I samples a softer region of the PES that approaches the basin of an alternative low-energy motif, motivating the minimum-energy pathway analysis between isomers I and III discussed below.

The magnitude of nuclear motion is quantified using the RMSD of all 15 atomic positions relative to the optimized reference geometry, after removal of overall rotation and translation (Figure 1b). For each trajectory frame, the RMSD is computed as the root-mean-square displacement over all atoms; the values reported below correspond to the time average and standard deviation along the production trajectory. At 100 K, isomer II remains close to its optimized geometry, with a time-averaged RMSD of 0.073 ± 0.014 Å. In contrast, isomer I exhibits substantially larger distortions at this temperature, with a time-averaged RMSD of 0.394 ± 0.042 Å, indicating that this structure samples a broader region of configurational space under the same thermal conditions. At 300 K, isomer II shows a clear increase in structural mobility (0.181 ± 0.028 Å), whereas isomer I maintains a similar time-averaged RMSD but develops a broader distribution of configurations (0.394 ± 0.053 Å). This behavior is consistent with a softer finite-temperature configurational landscape for isomer I, although the RMSD analysis alone does not distinguish whether the large-amplitude motion reflects continuous distortions or switching between distinct local coordination regimes.

The microscopic origin of the overall RMSD fluctuations can be rationalized by examining the evolution of individual Ti–O coordination distances. As shown in the right panel of Figure 1c, one specific Ti–O pair, hereafter denoted Ti_flux_–O_flux_, samples distances of up to ca. 3.0 Å at both 100 and 300 K, clearly exceeding typical nearest-neighbor Ti–O distances, which are usually between 1.6 and 2.2 Å. The relevance of this local coordinate was first suggested by geometry optimization of selected distorted AIMD snapshots: the snapshot displaying the largest Ti_flux_–O_flux_ separation relaxed to isomer III. This indicates that the Ti_flux_–O_flux_ distance is a useful descriptor of the structural connection between isomers I and III.

The temperature dependence, however, is decisive. At 100 K, the trajectory remains largely confined to the long-distance Ti_flux_–O_flux_ regime, with only limited fluctuations around this elongated coordination motif. At 300 K, by contrast, the system repeatedly samples both shorter and longer Ti_flux_–O_flux_ distances, giving rise to back-and-forth switching between two distance regimes along the trajectory. To quantify this fluxional sampling, we separated the 300 K Ti_flux_–O_flux_ distance distribution in all nuclear trajectories into short- and long-distance regimes using a cutoff of 2.55 Å, corresponding approximately to the minimum between the two populations in Figure 1c. With this definition, ca. 35% of the AIMD snapshots belong to the short-distance coordinated regime while ca. 65% sample the long-distance regime. This behavior is consistent with the RMSD analysis in Figure 1b, in which isomer I exhibits large-amplitude RMSD fluctuations already at 100 K, but broader and genuinely bimodal configurational sampling at 300 K. In contrast, the distribution of all Ti–O nearest-neighbor distances (Figure 1c, left) remains sharply peaked at ca. 2.0 Å at both temperatures, indicating that the rest of the coordination network remains comparatively rigid. This comparison shows that the large-amplitude motion is localized within a specific Ti–O subunit and its local coordination environment, rather than reflecting global softening of the nanocluster framework.

The reversible switching between short- and long-distance Ti_flux_–O_flux_ regimes at 300 K therefore identifies a key local descriptor of the fluxional motion. To test whether this local descriptor is associated with a physically meaningful pathway between low-energy motifs, we computed the minimum-energy pathway connecting isomer I to structurally related isomer III using the climbing-image nudged elastic band (CI-NEB) method^45^ with eight images, including six intermediate images. The pathway features an activation barrier of ca. 0.4 eV relative to isomer I. Refinement of the highest-energy NEB image by the dimer method,^46^ followed by frequency analysis, confirmed a first-order saddle point (see section S2). The associated imaginary mode contains a significant contribution from the local Ti–O rearrangement identified above, consistent with its involvement in the isomer I–III pathway.

Although this barrier is substantially larger than *k*_B_ *T* at 300 K, this comparison refers to only the thermal energy per degree of freedom. The (TiO_2_)_5_ cluster contains 39 vibrational degrees of freedom, and energy redistribution among coupled anharmonic modes can transiently drive collective excursions along the relevant local coordination coordinate. This interpretation is consistent with the AIMD trajectories. At 300 K, isomer I repeatedly switches between short- and long-distance Ti_flux_–O_flux_ regimes associated with excursions toward the isomer III region of configurational space, whereas at 100 K, no comparable back-and-forth switching is observed.

To further test whether the Ti_flux_–O_flux_ elongation reflects a collective finite-temperature motion, we analyzed the 300 K AIMD trajectory using the velocity-covariance mode approach of Strachan.^47^ The leading dynamical mode involves concerted displacement of the Ti_flux_–O_flux_ pair together with its local coordination environment, reproducing the short–long Ti_flux_–O_flux_ rearrangement identified from the distance distributions and NEB pathway. This confirms that the selected Ti_flux_–O_flux_ distance is a useful structural descriptor of a broader multiatom fluxional motion, rather than a complete one-dimensional reaction coordinate. Details of the dynamical mode analysis are provided in section S3.

Having established that the 300 K trajectory activates a collective fluxional mode that is described well by the Ti_flux_–O_flux_ distance, we next ask whether this structural motion has direct electronic consequences. This is the central point of the study. If fluxionality merely increases structural disorder in the nanocluster, its photophysical relevance would be limited; if, however, this motion reorganizes the low-energy excited-state manifold, then fluxionality becomes a direct structural mechanism for modulating nonadiabatic dynamics.

The connection between structure and excitation energy is shown in Figure 2a. The time evolution of the S_1_ excitation energy closely follows the Ti_flux_–O_flux_ distance, revealing a pronounced coupling between this local coordination distortion and the electronic structure. Quantitatively, the anticorrelation between these values is pronounced (*R* ^2^ ≈ 0.67). Elongation of the Ti_flux_–O_flux_ pair lowers the S_1_ excitation energy. Similar trends are obtained for S_2_ and S_3_, indicating that this single local distortion accounts for a substantial fraction of the energetic modulation of the low-energy excited-state manifold.

**Figure 2:**
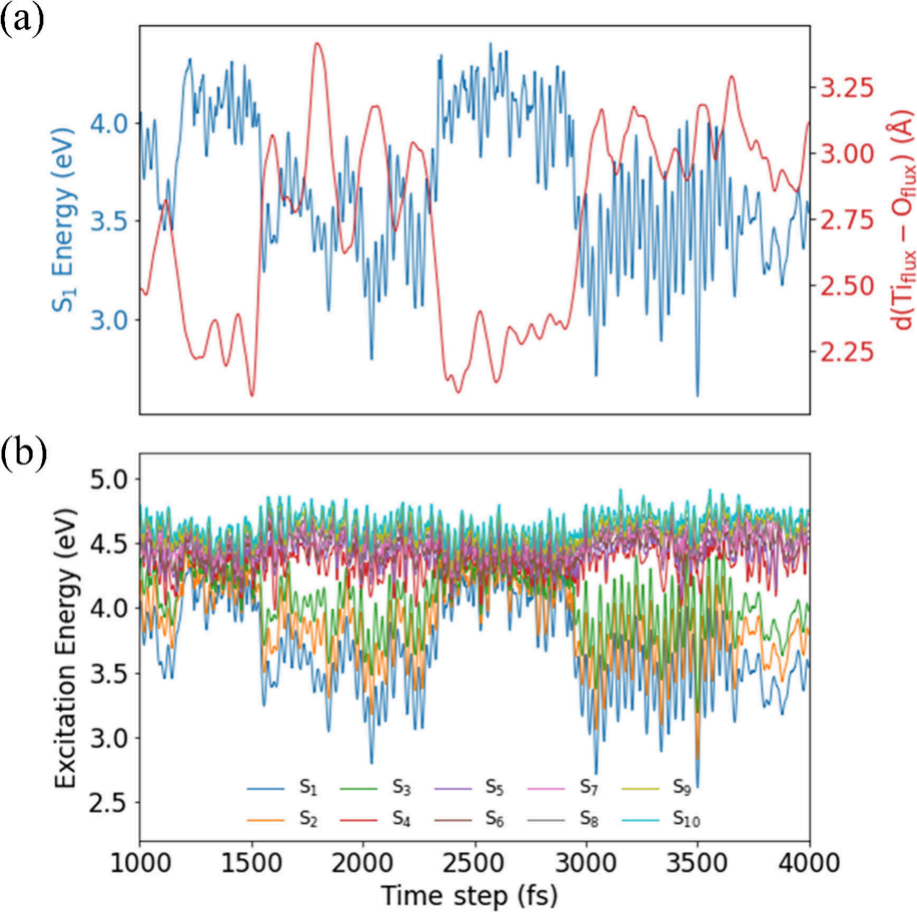
Coupling between fluxional nuclear motion and the low-energy excited-state manifold of isomer I at 300 K. (a) Comparison between the S_1_ excitation energy and the Ti_flux_–O_flux_ distance, used as a structural descriptor of the dominant fluxional mode. (b) Time evolution of the 10 lowest singlet excitation energies, S_1_–S_10_, along the AIMD trajectory.

This behavior is thus not restricted to an isolated electronic state. The three lowest excited states evolve in a strongly correlated manner (*R* ^2^ ≈ 0.9–0.95 (Figure 2b)), defining a coupled low-energy manifold that responds collectively to the fluxional coordinate. Therefore, the Ti_flux_–O_flux_ motion does not merely perturb S_1_ but reorganizes the relative energetic position of the low-lying excited-state manifold as a whole. In contrast, higher-lying excitations (S_4_–S_10_) show only weak to moderate correlation with the Ti_flux_–O_flux_ coordinate (*R* ^2^ < 0.3), despite remaining correlated among themselves. This suggests that the upper manifold is more strongly influenced by more delocalized or collective structural fluctuations, whereas the low-energy manifold is selectively controlled by the localized Ti_flux_–O_flux_ rearrangement.

NTO analysis of representative snapshots confirms that the low-energy excitations retain the characteristic O 2p → Ti 3d charge-transfer character of small titania clusters, while their spatial localization reorganizes along the fluxional segment of the trajectory (Figure S3, Supporting Information). Because S_1_–S_3_ are closely spaced in this region, the NTOs are interpreted qualitatively as evidence that the dominant charge-transfer excitation is redistributed among nearby low-lying states as the Ti_flux_–O_flux_ distance changes, rather than as strict adiabatic-state tracking. The persistence of O 2p → Ti 3d character, together with the geometry-dependent changes in localization, indicates that Ti_flux_–O_flux_ motion reshapes the low-energy excited-state manifold without generating a qualitatively unrelated excitation. Consistent with this interpretation, Hirshfeld charge analysis shows a reproducible redistribution of electron density within the fluxional Ti–O subunit between short- and long-distance Ti_flux_–O_flux_ configurations (Figure S4), supporting the assignment of this coordinate as an electronically active distortion.

The time evolution of the low-energy excited-state manifold in Figure 2a is reflected directly in the distribution of the S_1_–S_0_ energy gap (Figure 3a). At 100 K, the gap distribution is comparatively narrow and centered around ca. 3.5–3.7 eV, consistent with sampling being largely restricted to a nonswitching Ti_flux_–O_flux_ regime. The S_1_ excitation-energy distribution has a mean value of 3.55 ± 0.17 eV at 100 K. At 300 K, by contrast, where the Ti_flux_–O_flux_ coordination-switching motion becomes thermally activated, the distribution broadens markedly, with the S_1_ energy increasing to 3.68 ± 0.36 eV, and develops two distinct regions: a lower-gap component around ca. 3.3–3.6 eV and a higher-gap component extending around ca. 4.0–4.2 eV. Thus, activation of the fluxional Ti_flux_–O_flux_ motion approximately doubles the width of the S_1_ excitation-energy distribution. This bimodality is the electronic signature of fluxional sampling. Rather than reflecting simple thermal broadening around a single minimum, the activated Ti_flux_–O_flux_ coordinate partitions the low-energy excited-state landscape into two regimes associated with short- and long-distance local coordination environments.

**Figure 3:**
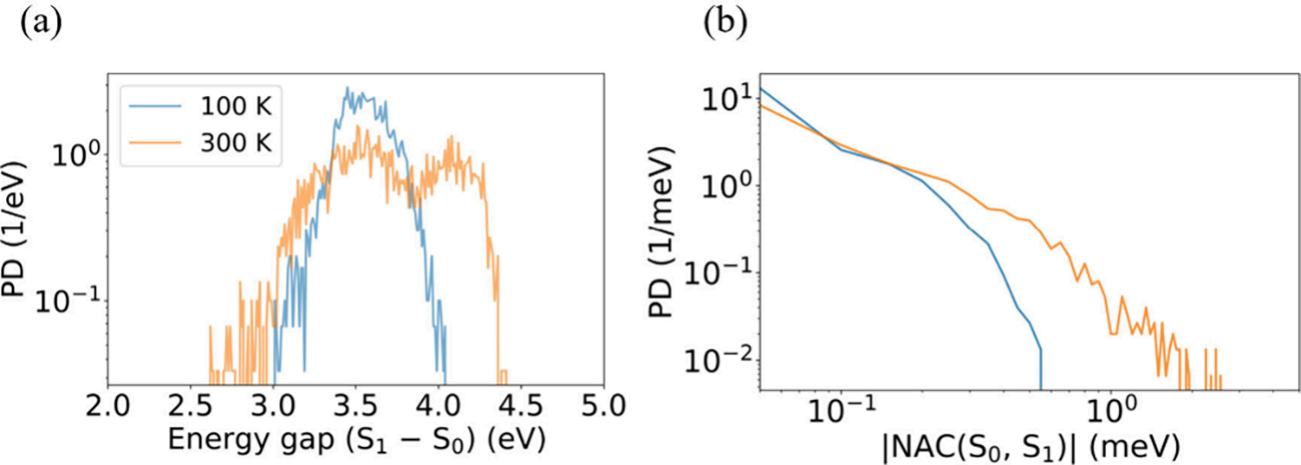
(a) Probability distribution of the S_1_–S_0_ energy gap sampled along the AIMD trajectories at 100 and 300 K. (b) Probability distribution of nonadiabatic coupling magnitude |NAC­(S_0_, S_1_)| at 100 and 300 K.

This restructuring of the energy gap landscape is accompanied by a change in ground-state coupling statistics (Figure 3b). In fact, the 300 K trajectory exhibits a broader |NAC­(S_0_, S_1_)| distribution, with a clearly enhanced high-coupling tail relative to 100 K. This increase is consistent with the nature of the fluxional motion. Repeated transitions between short- and long-distance Ti_flux_–O_flux_ configurations generate large-amplitude nuclear displacements along a coordinate that strongly modulates the low-energy excited-state manifold. Since nonadiabatic couplings measure the sensitivity of the electronic wave function to nuclear motion, configurations where the Ti_flux_–O_flux_ unit is rapidly reorganizing are expected to produce stronger nonadiabatic coupling between S_1_ and S_0_. Thus, fluxionality affects recombination-relevant quantities in two coupled ways. It broadens the instantaneous S_1_–S_0_ energy gap landscape and increases the probability of sampling geometries where nuclear motion efficiently mixes the lowest excited state with the ground state.

Before discussing the NA-MD results, we note that these simulations are performed within the NBRA framework using ground-state AIMD trajectories. Thus, possible feedback of the electronic excitation on the nuclear motion is not explicitly included. Inclusion of excited-state structural relaxation may quantitatively modify the computed relaxation and recombination time scales, although the exact direction of this change is dependent on the system. It may accelerate the dynamics if excited-state gradients drive the system toward geometries with smaller energy gaps between neighboring excited states and/or larger nonadiabatic couplings. Conversely, it may also slow relaxation if the excited-state PES topology favors geometrical evolution toward regions with larger energy gaps or weaker nonadiabatic couplings. Given that relaxation within the manifold of higher excited states is a relatively fast process in the present simulations, the inclusion of back-reaction is expected to have a comparatively limited effect on these time scales. For S_1_ → S_0_ recombination, the effect is less straightforward. Excited-state structural relaxation may enhance recombination if it increases the sampling of small-gap or high-coupling configurations, but it could also partially suppress recombination if the system relaxes into geometries with weaker coupling to the ground state. Therefore, the absolute relaxation and recombination times reported below should be interpreted within the NBRA approximation, and future non-NBRA simulations would be valuable for quantitatively assessing the role of excited-state back-reaction. Nevertheless, the qualitative mechanism identified here is expected to remain robust, because it originates from a thermally activated Ti_flux_–O_flux_ coordination-switching motion already sampled in the finite-temperature nuclear ensemble and strongly coupled to the low-energy excited-state manifold.

The changes in the S_1_–S_0_ gap and NAC distributions are directly reflected in the nonadiabatic dynamics (Figure 4). We first consider ground-state recombination starting from S_1_. Within FSSH, the recombination time is essentially unchanged upon heating, with τ values of 19.7 ± 2.8 ps at 100 K and 19.5 ± 1.7 ps at 300 K. Thus, within standard FSSH, no clear temperature dependence is observed for S_1_ → S_0_ recombination in this system. In contrast, the decoherence-corrected mSDM dynamics show a pronounced acceleration at 300 K: the recombination time decreases from 140.5 ± 9.9 ps at 100 K to 41.0 ± 7.0 ps at 300 K, corresponding to a reduction by a factor of approximately 3.4. This difference indicates that the enhanced sampling of high-NAC geometries at 300 K translates into faster net recombination only when electronic decoherence is included.

**Figure 4:**
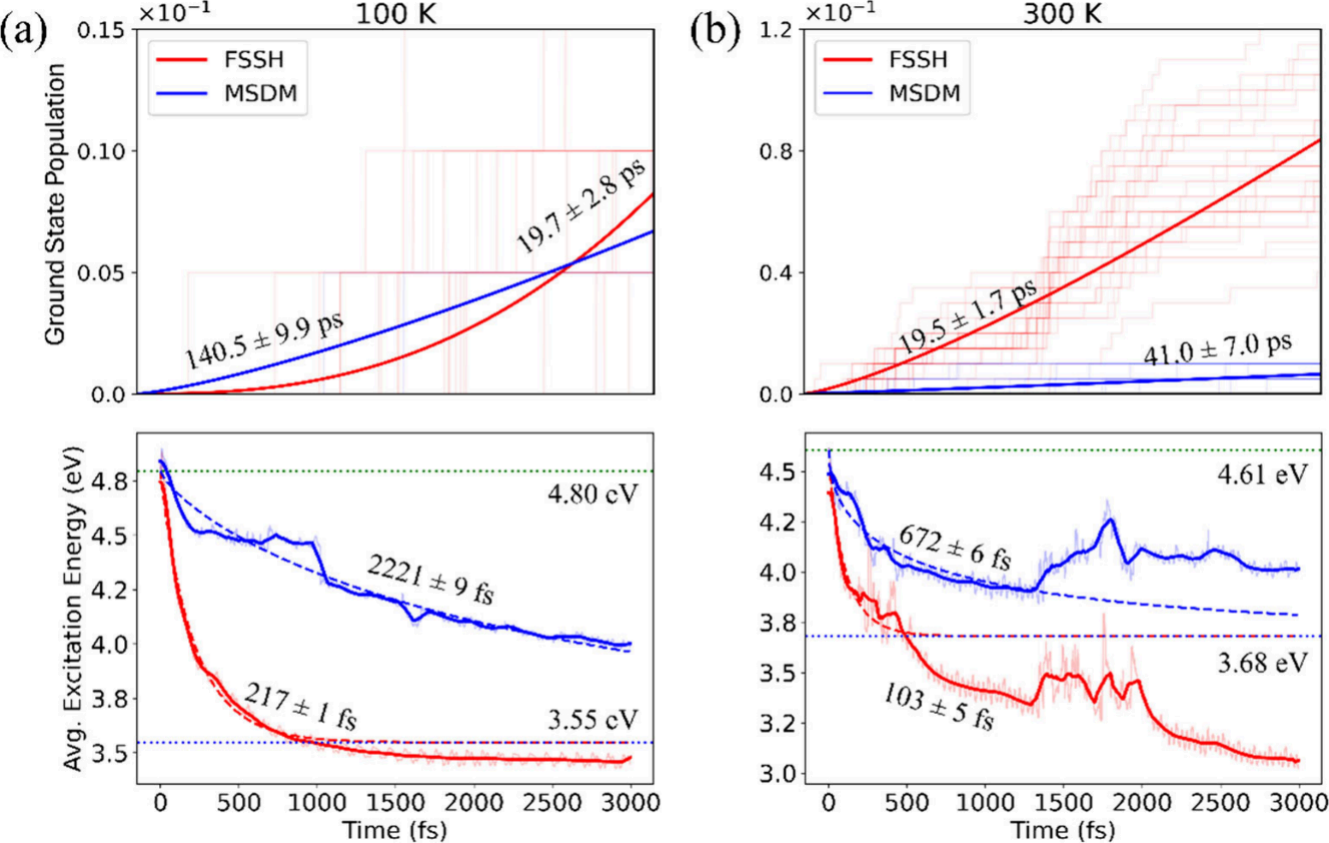
Temperature-dependent nonadiabatic dynamics of the fluxional isomer. Results at (a) 100 and (b) 300 K. Top panels show the growth of the ground-state population after initialization in S_1_. Lighter step-like curves are individual trajectories/batches, while thick solid curves are averaged ground-state populations; the annotated values correspond to the fitted population-growth time scales. Bottom panels show the decay of the average excitation energy after initialization in a higher bright excited state. Lighter curves are raw averaged energy traces, thick solid curves smoothed averages, and dashed curves stretched-exponential fits to the early time relaxation. Dotted horizontal lines indicate the energy of the initially excited state/ensemble reference (green) and the S_1_ reference energy (blue). Red and blue denote FSSH and mSDM, respectively.

This behavior can be rationalized by considering the different role of coherence in the two approaches. In FSSH, long-lived electronic coherence allows repeated forward and backward population exchange between S_1_ and S_0_, so that stronger instantaneous couplings do not necessarily produce faster net recombination. In mSDM, nuclear motion-induced decoherence suppresses such back-transfer, making individual nonadiabatic events more effectively irreversible. The broader 300 K |NAC­(S_0_, S_1_)| distribution, including its enhanced high-coupling tail, therefore leads to more efficient net population transfer from S_1_ to S_0_ in mSDM. Importantly, this acceleration is not driven by a simple reduction of the S_1_–S_0_ gap. The gap distribution of 300 K is broader and more heterogeneous than that at 100 K, but it does not shift uniformly toward smaller gaps. Fluxionality therefore affects recombination statistically, by increasing the probability of visiting geometries with favorable coupling conditions.

The relaxation dynamics within the excited-state manifold shows a related, but mechanistically distinct, temperature dependence. In these simulations, the dynamics is initialized from the same bright higher-energy state, S_9_, and relaxation is monitored through the decay of the average excitation energy toward the S_1_ region. Expectedly, relaxation is faster at 300 K than at 100 K for both methods. With the FSSH method, the relaxation time decreases from 217 ± 1 fs at 100 K to 103 ± 5 fs at 300 K, making relaxation approximately 2 times faster. With the mSDM method, the relaxation time decreases from 2221 ± 9 fs at 100 K to 672 ± 6 fs at 300 K, corresponding to an acceleration by a factor of ca. 3.3. This acceleration cannot be assigned primarily to a systematic increase in adjacent-state NACs, since the distributions of |NAC­(S_*i*_, S_*i*+1_)| at 100 and 300 K show comparable magnitudes and overlapping probability ranges, without a uniform increase at 300 K (Figure S5). Instead, the faster relaxation at 300 K reflects the fluxional reshaping of the excited-state energy landscape. The relevant quantity is the energetic separation between the initially populated bright state and the S_1_ destination. At 100 K, the initial S_9_ energy is around 4.80 eV and the S_1_ reference lies at ∼3.55 eV, requiring dissipation of approximately 1.25 eV. At 300 K, the corresponding values are ca. 4.61 and 3.68 eV, reducing the relaxation-energy window to approximately 0.93 eV. Thus, at 300 K, the initially populated bright state lies closer in energy to the S_1_ region, so less excess excitation energy must be dissipated during relaxation. Accounting for decoherence effects slows the relaxation dynamics, as seen by comparison of the mSDM and FSSH energy decay dynamics (Figure 4).

This interpretation is supported by a subset-resolved analysis of the 300 K trajectory (Figure S6). Initial conditions were separated according to the instantaneous S_1_ reference energy sampled along the trajectory, giving two subsets: a high-S_1_-energy subset with an average S_1_ energy of 4.09 eV and a low-S_1_-energy subset with an average S_1_ energy of 3.46 eV. Relaxation is faster in the high-S_1_-energy subset, where the energy gap between the initially populated bright state and the S_1_ destination is smaller. Following the FSSH method, the relaxation time increases from 70 ± 2 fs in the high-S_1_-energy subset to 147 ± 1 fs in the low-S_1_-energy subset, while with the mSDM method, it increases from 138 ± 3 to 1564 ± 83 fs. The relaxation times obtained from the full 300 K ensemble lie between the corresponding subset-specific limits, consistent with the full trajectory sampling both types of local excited-state energy landscapes.

Taken together, these results point to a unified mechanistic picture in which fluxional nuclear motion at 300 K continuously modulates the electronic structure, giving rise to a broadened and heterogeneous excited-state landscape. Rather than uniformly decreasing energy gaps, fluxionality increases the likelihood of accessing structural configurations that are dynamically favorable for nonadiabatic transitions, particularly those associated with strong coupling to the ground state. In decoherence-corrected dynamics, these configurations act as efficient and irreversible decay channels, leading to accelerated relaxation and recombination. In contrast, within FSSH, the same coupling events are partially compensated by coherent back-transfer, masking their net effect and yielding similar recombination times at both temperatures, highlighting the key role of decoherence in these processes.

The results presented here demonstrate that structural fluxionality is not only a finite-temperature structural effect but also a mechanism for controlling excited-state behavior in oxide nanoclusters. In the low-energy (TiO_2_)_5_ isomer studied here, finite-temperature AIMD, TD-DFT excitation analysis, and NA-MD identify a collective fluxional mode, conveniently represented by the Ti_flux_–O_flux_ distance, as the key structural coordinate controlling the electronic response. Motion along this coordinate becomes pronounced at 300 K, where the NC repeatedly samples short- and long-distance Ti_flux_–O_flux_ structural configurations, while such switching is suppressed at 100 K. Transition-state searches using the NEB method further confirm that this local rearrangement is connected to a minimum-energy pathway between structurally related low-energy motifs.

The central finding is the direct and selective link between localized fluxional nuclear motion and the low-energy excited-state response. Elongation of the Ti_flux_–O_flux_ distance modulates the low-energy excited-state manifold, with the S_1_ excitation-energy distribution increasing from 3.55 ± 0.17 eV at 100 K to 3.68 ± 0.36 eV at 300 K. Rather than reflecting random thermal disorder, this modulation follows a specific coordination coordinate and gives rise to distinct excitation-energy environments within the 300 K ensemble. Natural transition orbital and Hirshfeld charge analyses support this picture, showing that the low-energy excitations retain the characteristic O 2p → Ti 3d charge-transfer character of titania clusters while their spatial localization and charge distribution change along the fluxional coordinate.

These structural and electronic effects have direct consequences for nonadiabatic dynamics. Fluxionality does not simply shrink the average energy gap. Instead, its influence is statistical. The 300 K ensemble samples broader and more heterogeneous distributions of excitation energies and |NAC­(S_0_, S_1_)|, increasing the probability of visiting molecular geometries with stronger ground-state coupling. Relaxation toward the low-energy excited-state region is accelerated at 300 K because the initially populated bright state lies closer in energy to the S_1_ destination, reducing the amount of excess excitation energy that must be dissipated. Ground-state recombination is also accelerated in decoherence-corrected dynamics because geometries with stronger S_1_/S_0_ nonadiabatic coupling become more effective net decay channels when coherent back-transfer is suppressed.

Overall, these findings establish structural fluxionality as an active descriptor of excited-state behavior in oxide NCs. The key effect is not a perturbative broadening around a static minimum but the emergence of heterogeneous and multimodal distributions of electronic properties generated by thermally accessible coordination dynamics. In such systems, excited-state relaxation and recombination are governed by the interplay among nuclear motion, energy gap fluctuations, nonadiabatic coupling statistics, and decoherence, rather than by any single static descriptor such as an optimized geometry or average excitation energy. Similar fluxionality-driven effects may therefore be widespread across subnanometer oxide, metal oxide, and heterogeneous photocatalytic systems, where controlling local coordination flexibility could offer a route to tune excited-state lifetimes and photophysical function.

## Supplementary Material



## Data Availability

Python scripts, inputs, and key outputs are available via Zenodo (10.5281/zenodo.20737596).^48^

## Notes

Aditional computational details, NEB and transition-state refinement (Figure S1), displacement vectors of the Stachan-V mode (Figure S2), natural transition orbitals along the fluxional trajectory (Figure S3), time evolution of the Hirschfeld charges (Figure S4), adjacent-state nonadiabatic couplings (Figure S5), and basin-resolved relaxation dynamics (Figure S6) (PDF)
