# Supplementary material for: Thermally Activated Fluxionality Accelerates Nonradiative Decay in Titania Nanoclusters
Source: J Phys Chem Lett. 2026 Jun 30;17(28):7907–15. doi: 10.1021/acs.jpclett.6c01707 (PMC13383825; doi:10.1021/acs.jpclett.6c01707)
Supplement: Supplementary file 1 [file jz6c01707_si_001.pdf]

Supporting Information for:

Thermally Activated Fluxionality Accelerates Nonradiative Decay in Titania Nanoclusters

Miguel Recio-Poo,[a] Stefan T. Bromley,[a,b] Scott G. Sayres,[c,d] Francesc Illas,[a] Alexey V. Akimov,[e]* Ángel Morales-García[a]*

[a] *Departament de Ciència de Materials i Química Física & Institut de Química Teòrica i Computacional (IQTCUB), Universitat de Barcelona, 08028 Barcelona, Spain*

[b] *Institució Catalana de Recerca i Estudis Avançats (ICREA), 08010 Barcelona, Spain*

[c] *School of Molecular Sciences, Arizona State University, Tempe, AZ 85287, United States*

[d] *Biodesign Center for Applied Structural Discovery, Arizona State University, Tempe, AZ 85287, United States*

[e] *Department of Chemistry, University at Buffalo, The State University of New York, Buffalo, NY 14260, United States*

E-mail: alexeyak@buffalo.edu; angel.morales@ub.edu

Table of Content

S1. Computational details **S2**

S2. NEB and TS refinement **S4**

S3. Dynamical mode analysis **S5**

S4. NTOs along the fluxional trajectory **S7**

S5. Hirshfeld charges analysis **S8**

S6. NACs between adjacent states **S10**

S7. Basin-resolved relaxation dynamics **S11**

S1. Computational details

Ground-state electronic structure calculations and AIMD simulations were performed within Kohn–Sham DFT using the PBE exchange–correlation functional[1] as implemented in CP2K.[2] GTH pseudopotentials together with DZVP-MOLOPT-SR-GTH (Ti) and DZVP-MOLOPT-GTH (O) basis sets were employed.[3] The auxiliary plane-wave basis used a cutoff of 300 Ry and a relative cutoff of 50 Ry. Dispersion interactions were included through the Grimme DFT-D3 correction in its original zero-damping formulation with PBE reference parameters.[4] Additional AIMD details are provided in the main text.

Structural mobility along the AIMD trajectories was quantified using RMSDs of atomic positions after removal of overall translation and rotation. Selected Ti–O distance distributions were then used to identify local coordination changes responsible for the large-amplitude motion. To assess whether the dominant Ti–O rearrangement corresponds to a dynamically active collective motion, the 300 K trajectory of isomer I was analyzed using a velocity-covariance finite-temperature mode analysis following the Strachan approach.[5] This analysis identifies collective motions actively sampled along the trajectory, rather than harmonic normal modes around a single optimized geometry (see Section S3).

Vertical excitation energies were computed using linear-response TD-DFT within the TD-DFPT formalism in CP2K. A range-separated CAM-B3LYP-type functional was employed,[6] together with explicit Hartree–Fock exchange evaluated using truncated Coulomb operators. DZVP-MOLOPT-PBE0-type basis sets[7] and corresponding GTH pseudopotentials[8] were used for both elements. For each sampled structure, the lowest 20 singlet excited states were computed, including an extended virtual space of approximately 70–80 unoccupied orbitals to ensure a balanced description of the excitation manifold. Excited-state character was analysed for selected configurations using natural transition orbitals (NTOs), obtained by singular-value decomposition of the one-particle transition density matrix. NTOs provide a compact representation of the hole and particle components of each excitation and were used qualitatively to visualize changes in charge localization along representative fluxional segments.

Nonadiabatic molecular dynamics simulations were performed with Libra package[9,10] within the neglect-of-back-reaction approximation (NBRA)[11-13]. Electronic

amplitudes were propagated in the time-dependent adiabatic basis using local diabatization to stabilize electronic phases and enable robust integration of the time-dependent Schrödinger equation.

Two dynamical processes were analyzed: relaxation within the excited-state manifold and recombination to the ground state. Following the Kasha-type picture,[14] and consistent with our previous study of size-selected $(TiO_2)_n$ clusters,[15] initially populated higher excited states are expected to relax toward the low-energy excited-state manifold before experiencing a slower nonradiative decay to S_0. In the present simulations, relaxation times were extracted from the decay of the average excitation energy, whereas recombination times were obtained from the growth of the S_0 population.

Population transfer was simulated using standard fewest-switches surface hopping (FSSH)[16] and the decoherence-corrected modified simplified decay of mixing (mSDM) approach.[17] FSSH provides reference dynamics without explicit decoherence correction, whereas mSDM accounts for nuclear-motion-induced electronic decoherence and suppresses long-lived electronic superpositions and associated back-transfer. NA-MD trajectories were propagated for 3 ps using 1 fs timesteps for both nuclear and electronic degrees of freedom. Statistical sampling employed 30 independent nuclear initial conditions extracted along the AIMD trajectory, separated by 100 fs. For each initial condition, 500 stochastic surface-hopping realizations were performed. Observables were first averaged over stochastic realizations and then over initial conditions to obtain ensemble-averaged dynamics. Recombination simulations were initialized in S_1, and characteristic recombination times were obtained by fitting the ensemble-averaged growth of the ground-state population. Relaxation simulations were initialized in a higher-energy bright state, and relaxation times were obtained by fitting the decay of the ensemble-averaged excitation energy toward the corresponding S_1 reference energy. For the 300 K regime-resolved analysis, separate S_1 reference energies were used for the high- and low-S_1 regimes. Reported uncertainties were estimated from independent trajectory batches.

S2. NEB and transition-state refinement

The minimum-energy pathway connecting isomer 1 and isomer 3 was computed using the climbing-image nudged elastic band (CI-NEB) method. The initial path was constructed from the optimized endpoint geometries and discretized into eight images, including six intermediate images.

The highest-energy NEB image was subsequently refined using the dimer method to obtain a transition-state candidate. During refinement, the curvature along the dimer direction remained negative throughout the optimization, consistent with convergence toward a first-order saddle point. Final residual forces were reduced to ca. 0.05–0.09 eV $Å^{-1}$.

Vibrational analysis of the refined structure yielded a single imaginary frequency of approximately 147 cm^{-1}, while all remaining vibrational modes were real.

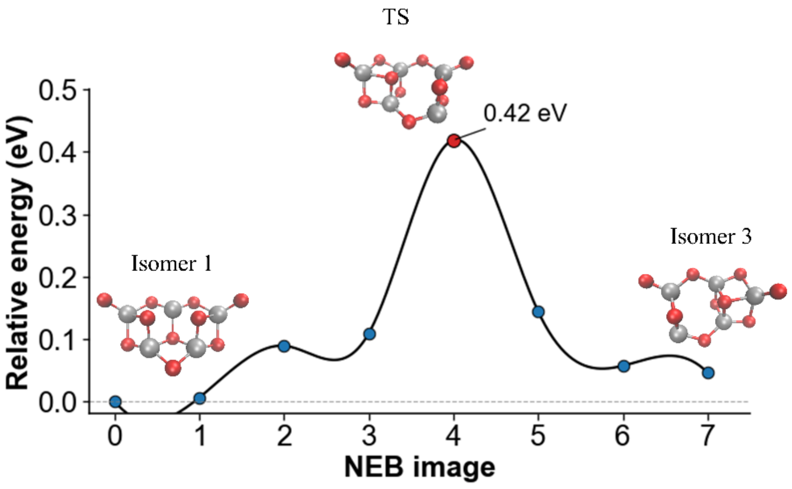

Figure S1. Minimum-energy pathway connecting isomers 1 and 3 of $(TiO_2)_5$ NC. The energy profile was obtained from a NEB calculation using eight images, including six intermediate images, and is reported relative to isomer 1. The highest-energy image was refined by the dimer method to locate the transition-state structure. Representative structures of isomer 1, the transition-state region, and isomer 3 are shown along the pathway. Grey and red spheres denote Ti and O atoms, respectively.

S3. Dynamical Mode Assignment of the Fluxional Coordinate

To assess whether the Ti_{flux}–O_{flux} elongation corresponds to a dynamically active collective motion, the 300 K AIMD trajectory of isomer I was analyzed using the velocity-covariance finite-temperature mode approach of Strachan.[18]. Prior to the analysis, overall translation and rotation were removed. The method constructs collective modes from velocity correlations sampled along the finite-temperature trajectory and therefore emphasizes motions that are dynamically active during the actual AIMD evolution.

This choice is important for the present system. The fluxional process involves anharmonic motion across a broad region of configurational space, rather than small-amplitude vibrations around a single equilibrium geometry. Standard normal-mode analysis would only describe the local harmonic curvature around an optimized structure and would therefore not capture the finite-temperature rearrangement sampled during AIMD. Quasi-harmonic and covariance-based structural fluctuation analyses can provide useful information on the dominant positional variance of a trajectory, but they do not necessarily distinguish whether a large-amplitude displacement corresponds to a dynamically active, velocity-correlated motion. In contrast, the Strachan-V approach directly identifies collective nuclear motions that are executed along the trajectory, making it more appropriate for assigning the fluxional coordinate considered here.

The leading Strachan-V mode involves a concerted displacement of the Ti_{flux}–O_{flux} subunit together with its local coordination environment (Figure S2a). This displacement pattern reproduces the short–long Ti_{flux}–O_{flux} rearrangement identified from the Ti–O distance distributions and the NEB pathway. The absolute mode index is method-dependent and should only be interpreted within this velocity-covariance analysis; the relevant result is the displacement pattern, which supports the assignment of the Ti_{flux}–O_{flux} distance as a useful structural descriptor of a broader multi-atom fluxional motion. The relative Strachan-V mode contributions are shown in Figure S2b.

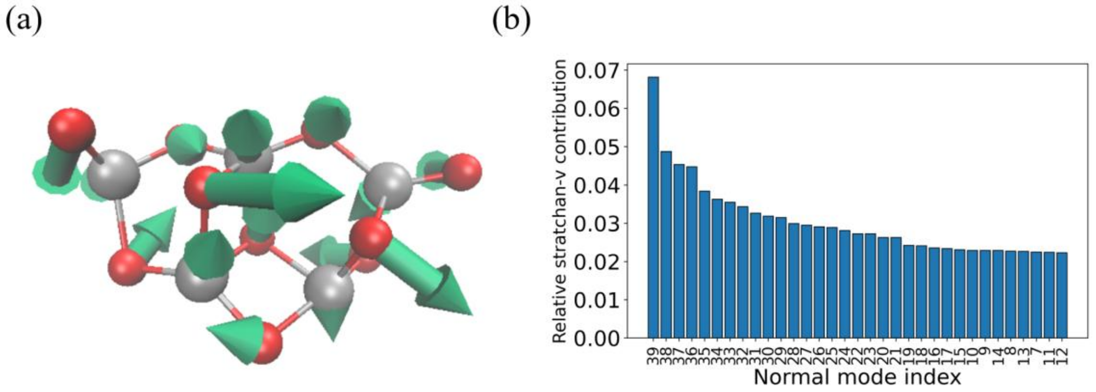

Figure S2. (a) Displacement vectors associated with the leading Strachan-V mode, showing a concerted motion involving the Ti_{flux}–O_{flux} subunit and its local coordination environment. (b) Relative contributions of the Strachan-V modes, normalized by the total contribution. The leading mode corresponds to mode 39 in the adopted indexing scheme and captures the localized collective rearrangement associated with the compact–elongated Ti_{flux}–O_{flux} coordinate.

S4. Natural transition orbital analysis along the fluxional trajectory

To examine how the electronic character evolves along the fluxional coordinate, we analysed natural transition orbitals (NTOs) for representative snapshots extracted from the 300 K AIMD trajectory in the time interval where the low-energy excitation energies vary most strongly. The NTOs show that the relevant low-energy excitations retain the characteristic O 2p → Ti 3d charge-transfer character expected for small titania clusters. However, the spatial localization of both hole and particle components changes along the trajectory as the local Ti–O coordination environment rearranges. Because S_1, S_2, and S_3 are closely spaced in this region, the adiabatic state labels should not be interpreted as strictly preserving a single electronic character at every timestep. Abrupt changes in the appearance of the NTOs are consistent with trivial crossings or strong adiabatic mixing within the low-energy excited-state manifold. Therefore, the NTO analysis is used here qualitatively to confirm that the fluxional motion reorganizes the spatial character of the low-energy charge-transfer excitations, rather than to assign a unique diabatic state along the full trajectory.

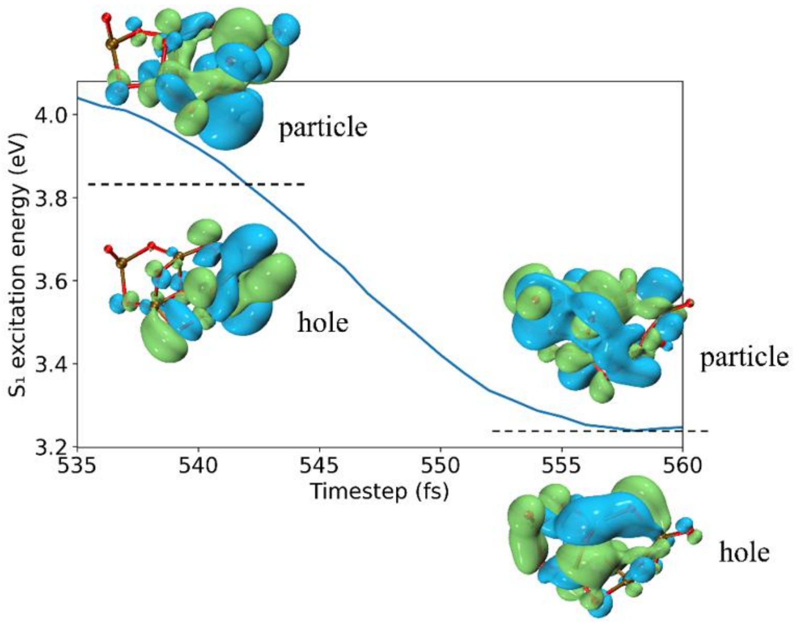

Figure S3. Representative natural transition orbitals (NTOs) along a fluxional segment of the 300 K AIMD trajectory of isomer 1. The selected snapshots span a pronounced modulation of the low-energy excitation energies. Hole and particle NTOs are shown below and above the energy curve, respectively. The NTOs retain the characteristic O 2p → Ti 3d charge-transfer character of titania clusters, while their localization patterns reorganize as the local Ti–O coordination environment evolves. Isosurface values are ±0.01 $Bohr^{-3}$.

S5. Hirshfeld charges analysis

Hirshfeld atomic charges were analyzed along the 300 K AIMD trajectory to assess whether the Ti_{flux}–O_{flux} rearrangement is accompanied by local charge redistribution. Because absolute atomic charges depend on the partitioning scheme, the analysis focuses on relative variations along the trajectory. Compared with chemically similar spectator atoms, Ti_{flux} remains systematically more positive, consistent with its undercoordinated local environment, while O_{flux} exhibits the largest charge variability among the oxygen atoms analyzed. The O_{flux} charge separates into two recurrent regimes that follow the compact and elongated Ti_{flux}–O_{flux} configurations: when O_{flux} approaches Ti_{flux}, it becomes less negative, whereas in the elongated configuration it recovers a more negative character. These trends indicate that the fluxional coordination change is accompanied by a reproducible local polarization response. This charge redistribution is consistent with the observed sensitivity of the low-energy excitation energies to the Ti_{flux}–O_{flux} coordinate, although it should be interpreted as a supporting descriptor rather than as a standalone mechanistic proof.

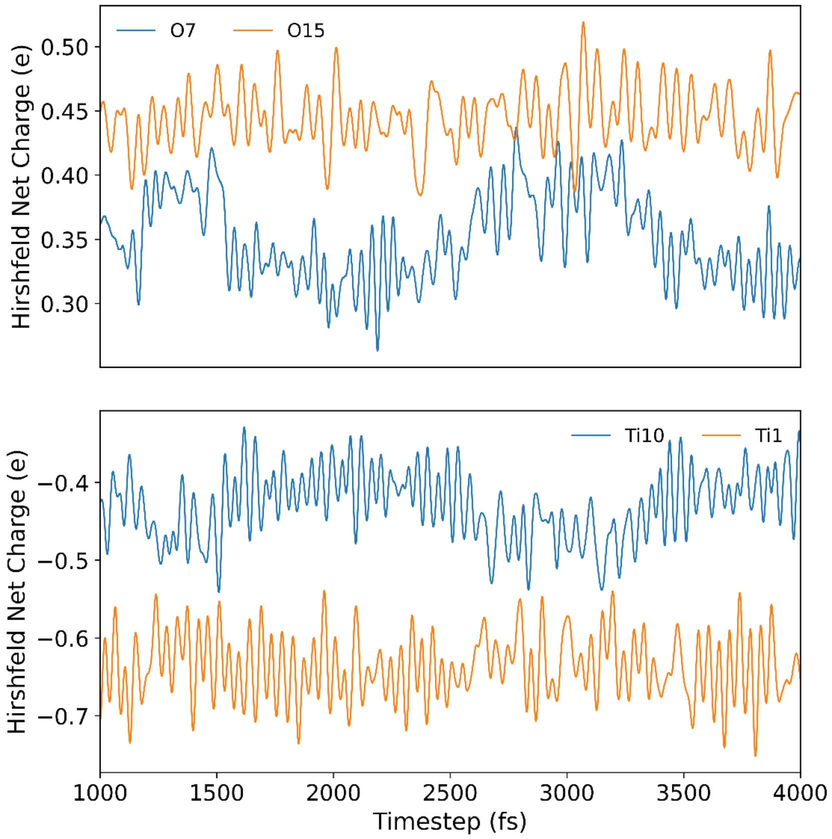

Figure S4. Time evolution of Hirshfeld net atomic charges along the 300 K AIMD trajectory. Top panel: comparison between oxygen atoms O_7 (fluxional site) and O_{15}

(reference oxygen). Bottom panel: comparison between titanium atoms Ti_{10} (fluxional site) and Ti_1 (reference titanium). Charges are reported in units of elementary charge (e) for each simulation timestep.

S6. NACs between adjacent states

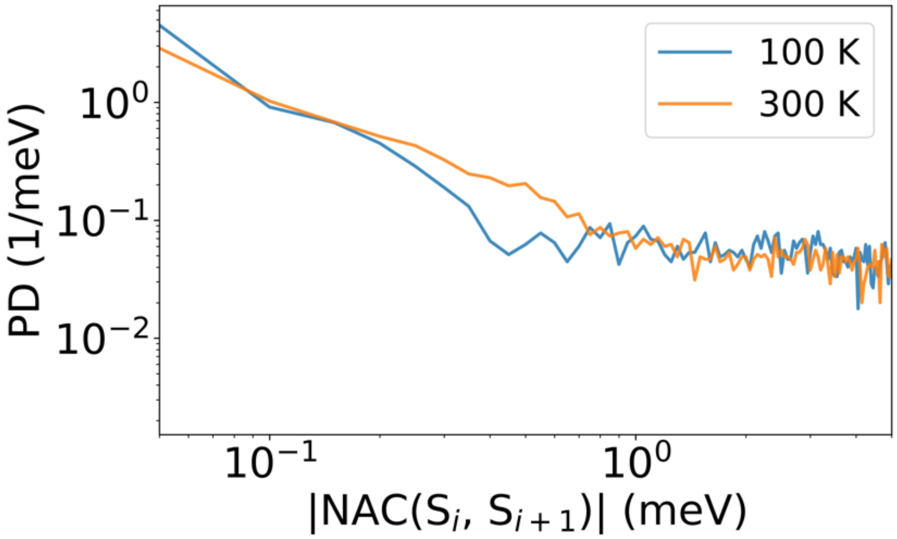

Figure S5. Probability distributions of adjacent-state nonadiabatic coupling magnitudes, $|\ \mathbf{NAC}(\mathbf{S_i}, \mathbf{S_{i+1}})\ |$, sampled along the 100 and 300 K trajectories of isomer 1.

S7. Basin-resolved relaxation dynamics

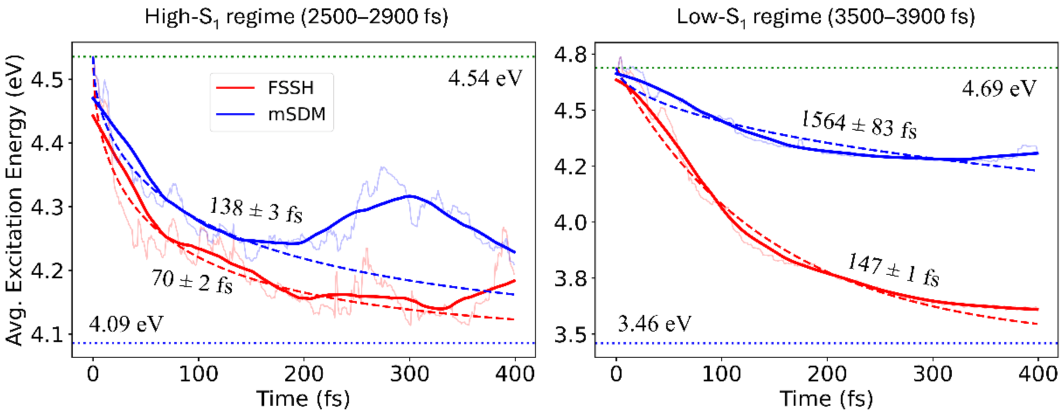

Figure S6. Basin-resolved relaxation dynamics for the 300 K trajectory of isomer 1. Initial conditions were separated into two regions of the fluxional trajectory: the high-S_1 regime (2500–2900 fs; left) and the low-S_1 regime (3500–3900 fs; right). Pale curves show raw averaged excitation-energy traces, thick solid curves show smoothed averages, and dashed curves show stretched-exponential fits to the early-time relaxation. Dotted horizontal lines indicate the initially bright excited-state reference energy (green) and the S_1 reference energy (blue). Red and blue denote FSSH and mSDM, respectively; annotated values are the fitted relaxation times.

References:

1 Perdew, J. P.; Burke, K.; Ernzerhof, M. Generalized gradient approximation made simple. *Phys. Rev. Lett.* **1996**, *77*, 3865.

2 Kühne, T. D.; Iannuzzi, M.; Del Ben, M.; Rybkin, V. V.; Seewald, P. CP2K: An electronic structure and molecular dynamics software package-Quickstep: Efficient and accurate electronic structure calculations. *J. Chem. Phys.* **2020**, *152*, 194103.

3 VandeVondele, J.; Hutter, J. Gaussian basis sets for accurate calculations on molecular systems in gas and condensed phases. *J. Chem. Phys.* **2007**, *127*, 114105.

4 Grimme, S.; Antony, J.; Ehrlich, S.; Krieg, H. A consistent and accurate ab initio parametrization of density functional dispersion correction (DFT-D) for the 94 elements H-Pu. *J. Chem. Phys.* **2010**, *132*, 154104.

5 Strachan, A. Normal modes and frequencies from covariances in molecular dynamics or Monte Carlo simulations. *J. Chem. Phys.* **2004**, *120*, 1-4.

6 Yanai, T.; Tew, D. P.; Handy, N. C. A New Hybrid Exchange-Correlation Functional Using the Coulomb-Attenuating Method (CAM-B3LYP). *Chem. Phys. Lett.* **2004**, *393*, 51−57.

7 Van de Vondele, J.; Hutter, J. Gaussian basis sets for accurate calculations on molecular systems in gas and condensed phases. *J. Chem. Phys.* **2007**, *127*, 114105.

8 Goedecker, S.; Teter, M.; Hutter, J. Separable dual-space Gaussian pseudopotentials. *Phys. Rev. B* **1996**, *54*, 1703.

9 Akimov, A. V. Libra: An Open-Source “Methodology Discovery” Library for Quantum and Classical Dynamics Simulations. *J. Comput. Chem.* **2016**, *37*, 1626-1649.

10 Shakiba, M.; Smith, B.; Li, W.; Dutra, M.; Jain, A.; Sun, X.; Garashchuk, S.; Akimov, A. Libra: A Modular Software Library for Quantum Nonadiabatic Dynamics. *Softw. Impacts* **2022**, *14*, 100445.

11 Craig, C. F.; Duncan, W. R.; Prezhdo, O. V. Trajectory surface hopping in the time-dependent Kohn-Sham approach for electronuclear dynamics. *Phys. Rev. Lett.* **2005**, *95*, 163001

12 Duncan, W. R.; Craig, C. F.; Prezhdo, O. V. Time-domain ab initio study of charge relaxation and recombination in dye-sensitized TiO_2. *J. Am. Chem. Soc.* **2007**, *129*, 8528−8543.

13 Prezhdo, O. V.; Duncan, W. R.; Prezhdo, V. V. Photoinduced electron dynamics at the chromophore−semiconductor interface: A time-domain ab initio perspective. *Prog. Surf. Sci.* **2009**, *84*, 30−68.

14 Kasha, M. Characterization of Electronic Transitions in Complex Molecules. *Discuss. Faraday Soc.* **1950**, *9*, 14−19.

15 Recio-Poo, M.; Shakiba, M.; Illas, F.; Bromley, S. T.; Akimov, A. V.; Morales-Garcia, A´. Hydration Accelerates Radiative and Nonradiative Recombination in Small TiO_2 Nanoclusters. *J. Phys. Chem. C* **2025**, *129*(3), 1806−1823.

16 Tully, J. C. Molecular dynamics with electronic transitions. *J. Chem. Phys.* **1990**, *93*, 1061−1071.

17 Smith, B.; Akimov, A. V. A comparative analysis of surface hopping acceptance and decoherence algorithms within the neglect of back-reaction approximation. *J. Chem. Phys.* **2019**, *151*, No. 124107.

18 Strachan, A. Normal modes and frequencies from covariances in molecular dynamics or Monte Carlo simulations. *J. Chem. Phys.* **2004**, *120*(1), 1-4.
